# Barriers and facilitators to fulfilling the teaching assistant role from nursing students’ perspective: a qualitative study

**DOI:** 10.1186/s12912-023-01645-7

**Published:** 2024-01-12

**Authors:** Shahzad Pashaeypoor, Zahra Amrollah Majdabadi, Ali Khanipour-Kencha, Nasrin Nikpeyma

**Affiliations:** 1 Department of Community Health and Geriatric Nursing, School of Nursing and Midwifery, Tehran University of Medical Sciences, Tehran, Iran; 2 Department of Medical-Surgical Nursing, School of Nursing and Midwifery, Tehran University of Medical Sciences, Tehran, Iran; 3 Department of Community Health and Geriatric Nursing, School of Nursing and Midwifery, Tehran University of Medical Sciences, East Nosrat Street, Tohid Square, Tehran, 1419732171 Iran

**Keywords:** Teaching assistant, Nursing, Nursing student, Teaching role

## Abstract

**Background:**

Employing postgraduate students as Teaching Assistants (TA) has become a common practice in many higher education institutions and is part of a growing international trend for professional practice.

**Objective:**

This study aimed to determine the barriers and facilitators to fulfilling the teaching assistant role from nursing students’ perspective.

**Methodology:**

This qualitative-descriptive study was conducted in 2022 on teaching assistants in the Faculty of Nursing and Midwifery of Tehran University of Medical Sciences. 13 teaching assistants were selected by targeted sampling method with maximum variation. The inclusion criteria were 2nd-semester postgraduate nursing students and above, having experience as a teaching assistant, and willing to participate in the research. Data were collected through semi-structured individual interviews for 45 to 60 min until saturation was reached. Data analysis was done manually and using the framework analysis method with steps: Familiarization, Identifying a thematic framework, indexing, Charting, data synthesis, mapping, and interpretation. The trustworthiness of the study data was determined with the Lincoln and Guba criteria.

**Results:**

Barriers to fulfilling the TAs’ role were divided into three main categories with nine subcategories: (1) Not accepting the teaching assistant as a clinical instructor (2) not being prepared to accept the TA role, and (3) improper planning of the TA plan. Facilitators are divided into two main categories with five subcategories: (1) internal motivation, and (2) empowering TAs.

**Conclusion:**

To facilitate management processes in the field of education and to achieve educational goals, including improving the quality of education and better learning, planning and compiling instructions to create written job descriptions for teacher assistants should be done, also, with careful planning, steps should be taken to scientifically employ capable teacher assistants as young and motivated forces in education processes.

**Supplementary Information:**

The online version contains supplementary material available at 10.1186/s12912-023-01645-7.

## Introduction

It has become a common practice in many educational institutions in different countries to employ postgraduate students as teaching assistants (TA) [1]. A TA is a person with at least a bachelor’s degree studying at a higher level of education and facilitates other students’ learning under the supervision of a senior professor [2, 3]. A TA is like a bridge between a senior professor and students. He fulfills roles such as advising students, grading students’ assignments, managing the courses taken, and leading discussion groups [1]. Other benefits of TAs for educational institutions include paying low salaries and training socializing future faculty members [1]. Professors employing TAs will have more time to design lectures, compile textbooks, and participate in scientific conferences [2]. On the other hand, students who work as a TA will better understand the teaching and learning processes and the course material. In addition, they gain teaching experience, interpersonal skills, and skills such as public speaking, group work, and time management [1].

Despite its advantages, teaching assistant causes some problems for institutions and teaching assistants. A primary disadvantage is the decreased quality of education for undergraduate students because teaching assistants lack enough teaching experience and knowledge [3]. In addition, they often have less time to study their courses and learn professional knowledge [2]. Moreover, TAs may have challenges in communication skills, such as establishing professional relationships with colleagues and interacting with students. Finally, working under the supervision of their main professor, they have limited independence and lack an official status in the university [1].

Today, employing TAs in education has become a part of the growing international trend for professional practice [4]. In addition, training the teaching assistants is emphasized as an essential component for training future professors [5]. In particular, clinical areas, including nursing, are more appropriate for employing TAs [6, 7]. Students’ clinical education is an essential component of nursing education, and its quality depends on the ability of professors and teaching assistants [8]. By integrating theoretical and clinical knowledge, TAs have the main role in facilitating nursing students’ learning and increasing their skills for providing quality nursing care [9]. Since nursing TAs are role models for students and support them, they must have professional knowledge and skills such as coaching skills, familiarity with teaching methods [10], and creating an effective learning environment [11]. As a result, nursing schools need to prepare postgraduates with professional abilities as clinical educators. However, its realization requires nursing school policymakers and managers to improve the postgraduates’ clinical qualifications and abilities to teach as clinical instructors and remove barriers to empowering the nursing TAs [9].

Despite the important role that the nursing TAs have, they often do not receive enough training concerning their role, depend only on their personal experiences in this field [8], and are not well prepared for this task [12]. Therefore, training skilled and capable TAs requires providing the necessary prerequisites and teaching basic knowledge and skills to them [12]. In addition, it requires identifying existing barriers and facilitators and eliminating upcoming barriers and challenges. However, since postgraduate students perform the TA role, understanding their perspectives can provide the necessary knowledge to design effective measures for identifying the facilitators and removing the barriers to effectively implementing the TA role. One of the best methods for obtaining their perspectives is to conduct qualitative studies according to search results, existing studies have only focused on one aspect of the issue. For example, Alhija 2021 assessed the challenges of teaching assistants. Specifically, this study focused on the concerns of graduate teaching assistants (GTAs) and their challenges in learning how to improve their work performance [1]. The study by Nichols et al. in 2020 examines the use of undergraduate teaching assistants in classrooms and their impact on student learning outcomes. The study focuses on the benefits and challenges associated with using TAs, including increased student engagement, improved communication skills, and enhanced critical thinking skills. The study also identifies the need for proper training and support for TAs to ensure their effectiveness in the classroom [7]. therefore, according to the search results, no study has yet examined all aspects of teacher assistants and the facilitators and barriers to this issue from the perspective of individuals who have experienced teacher assistants and given the need to employ teacher assistants in various fields, especially in medical sciences, and the lack of research in this regard especially in the cultural and social context of Iran, it is essential to conduct an in-depth investigation of this issue. Therefore, this qualitative research investigates the barriers and facilitators to fulfilling the TA role from the nursing students’ perspective in Iran’s cultural and social context.

## Methodology

This study was conducted using a qualitative-descriptive method and framework analysis to Understand the TAs’ perspective on the barriers and facilitators to fulfilling the TA role in 2022 in Iran, Tehran University of Medical Sciences, Faculty of Nursing and Midwifery. The population includes all postgraduate nursing students. Inclusion criteria included postgraduate nursing students in the 2nd semester and above who had TA experience and were willing to participate in the research. Exclusion criteria included not being willing to participate in the study and withdrawing from further interviews. To get the different views of the participants about the barriers and facilitators to fulfilling the TA role, the maximum variation targeted sampling was used to select the participants. To this purpose, qualified students were selected from both genders (i.e., male and female) and different nursing departments (i.e., community health, internal surgery, children, and special).

The researcher referred to the faculty’s education department, received a list of qualified students, and then communicated with them and invited them to participate in the study. The participants’ selection was continued until the data saturation was obtained. Finally, a total of 13 students participated in this study.

We used two tools to collect data: (1) a demographic information questionnaire (including age, gender, and master’s orientation), and (2) an in-depth semi-structured individual and a face-to-face interview. Interviewers used an interview guide and note-taking to collect intended data. The semi-structured interview questions were formulated by researchers according to the research purpose. The questionnaire started with a general question, “What is your opinion about TAs’ position?” (Attached in the Supplementary material). The researcher guided all interviews.

After obtaining the research permission and selecting qualified participants (postgraduate nursing students in the 2nd semester and above who had experience as assistant teachers and taught clinical skills to undergraduate students under the supervision of a professor), the research objectives were explained to them, and they were assured that their information would remain confidential and that their participation was voluntary. Then, their informed consent was obtained, and interviews were conducted at the place suggested by the participants. The interviews lasted 45 to 60 min. The participants consented to record the interview contents using a digital audio recorder. In addition, interviewers took notes and recorded the participants’ reactions. Once the interviews were over, the recorded information was listened to accurately 2 to 3 times and typed in Microsoft Word. In addition, the notes’ quality was also assessed. This process was done identically for all interviews. Qualitative research requires the researcher to immerse himself in the data. Therefore, the typed data were reviewed and compared with the audio recordings to ensure the accuracy of the data. The interviews lasted three months, from February to May. The framework analysis was employed to analyze the data.

A hierarchical framework analysis approach was used to classify and organize data based on key themes, concepts, and classes [13]. The data analysis was performed as follows:

1. Familiarization: In this stage, the recorded interviews were heard, and the manuscripts and notes were studied several times to get a general view and immerse in the data. Reading manuscripts or listening to the recordings several times improved our understanding of the key ideas and recurring concepts.
2. Identifying a thematic framework: In this stage, the researcher discusses the meanings of important topics and the clear connection between ideas and concepts. After repeatedly studying and reviewing the data, concepts, and materials with similar meanings or those thematically related were placed in the same group.
3. Indexing: The unit or parts of the data relating to a specific topic or class are identified in this step. This stage includes reading the manuscripts obtained from the interviews and taking notes on the manuscripts’ contents. These manuscripts were classified into different themes and reviewed several times to provide an index of the research data. This step was performed manually without using the software.
4. Charting: After indexing the data based on the thematic framework, the data are summarized in thematic tables. In other words, a summary of the main data is placed in the table’s entries so that all the data can be seen concurrently and placed in a table for easy access to all the data (Table 1).
5. The data synthesis, mapping, and interpretation: All the definitions and concepts issued by the participants were examined, and combined, and their relationships were determined. Finally, the barriers and facilitators to fulfilling the TAs’ role were clarified [14].

**Table 1:** An example of the thematic charting

Main Categories		Sub Categories	Indexes
Barriers	Not being prepared to accept the TA role	The stress of accepting the TA role	“In the first days, even though I had already studied the material, I was worried I might not answer students’ questions, and I was not prepared for how to behave in such a circumstance. In addition, I was not prepared to communicate with students.” (P 4)

### Trustworthiness

Lincoln and Guba’s criterion (1985), with four dimensions of credibility, dependability, conformability, and transferability, were used to validate the research data [15].

The research data was validated after extensive examination, long communication with some participants in the meetings, and checking the findings obtained from the members. In addition, the data was peer-checked by other research team members. To increase the credibility of the data, it was tried to select the participants from students studying in different nursing groups. To ensure the reliability of the data, the information collected from individual interviews and notes was read carefully by one of the research colleagues, and the results were compared.

To increase the conformability of the research data, we do not interfere with the information provided by the participants. Transferability means the applicability of the research findings in other similar fields. To improve the transferability of the data, we provided a detailed and complete description of the data and background conditions.

### Findings

Overall, 13 nursing postgraduate students participated in this research as TAs. Most of them were between 20 and 30 years old, there were 9 female and 4 male participants and The orientation of 6 participants was community health nursing, 5 participants were in Medical-Surgical Nursing, and 2 were in Critical Care Nursing. After 11 interviews, the data got saturated. However, two additional interviews were conducted to ensure no new data was needed. The participants’ characteristics are presented in Table 2.

**Table 2:** The participants’ characteristics of the study

Characteristics		Number
Age	20–25 year	5
	26–30 year	5
	31–35 year	2
	More than 35year	1
Sex	Male	9
	Female	4
Major Nursing of the TAs	Medical-Surgical Nursing	5
	Community Health Nursing	6
	Critical care Nursing	2
Total		13

**Fig. 1:**
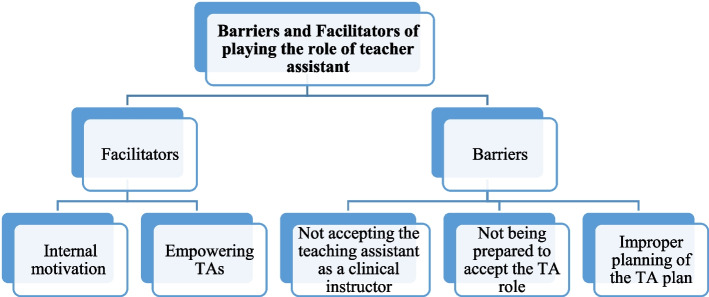
Conceptual map of barriers and facilitators of playing the role of teacher assistant

According to the framework analysis steps, the data were examined, and 196 indexes (initial code) were extracted. Then, indexes with similar thematic frameworks were placed in the thematic charting. In addition, a conceptual map was also presented to show the relationships between the concepts and present an overall plan (Fig. 1). Finally, the findings of analyzing the data obtained from interviewing the clinical assistants were divided into two main groups of barriers and facilitators:

A. Barriers to fulfilling TAs’ role were divided into three main categories with nine subcategories: (1) Not accepting the teaching assistant as a clinical instructor [not accepting the teaching assistant as a clinical instructor and non-cooperation of the clinical department personnel with them], (2) not being prepared to accept the TA role [the stress of accepting the TA role, lack of courses to familiarize TAs teacher their duties, insufficient supervision of the senior professor, and the chosen clinical filed not being the one interested by TA], (3) improper planning of the TA plan [the lack of a clear job description for TAs, the interference of TAs’ clinical training schedules with their educational schedules, and TAs’ inability to make decisions].
B. Facilitators fulfilling the TA role are also divided into two main categories with five subcategories as follows: (1) internal motivation [interest in being a TA, increasing self-confidence], (2) empowering TAs [practicing teaching skills, increasing theoretical and clinical knowledge, and perceived support from senior professor].

## Barriers to fulfilling the TA role

Barriers to fulfilling the TA role are divided into nine main categories with nine subcategories.

### Not accepting a TA as a clinical instructor

TAs stated that students and department personnel may not accept them for various reasons. However, some of these reasons include being young and having a small age gap with undergraduate students, insufficient experience managing students and answering their questions, a lack of clinical skills, and difficulty communicating with the clinical department personnel. This category is divided into two subcategories: students may not accept TAs as clinical instructors and clinical department personnel may not cooperate with them.

### Students may not accept TAs as clinical instructors

The participants said that if they were well-known instructors, students would further obey them and observe the rules and duties of the internship. They said the presence of an experienced instructor is necessary to acquire knowledge and clinical skills. For example, a participant said:

“*Students believe that senior instructors compared to a TA, can provide more and better information to them and also can learn more and better skills, and so they are reluctant to TAs.*” (P 9).

Another participant referred to the age difference between TAs and students:

“*I think the very small age difference between students and TAs makes them not perform well in the tasks and assignments given to them, and they were not taken seriously by students. In addition, they may not accurately observe the attendance and rest times*.” (P 10).

### Clinical department personnel may not cooperate with TAs

Some participants said that supervisors and staff of the clinical department may not behave properly with them because they are a student, and they may ask them to take care of patients. In addition, they may also misbehave with the students supervised by a TA. A participant explained the non-cooperation of personnel as follows:

“*The head nurses were not very cooperative, for example, if we wanted a class. We had difficulty coordinating the students’ visits from the echo department so that the students could see both the echo and the Cath Lab. For example, they refused us as a TA and insisted that we call our professors*.” (P 2).

### Not being prepared to accept the TA role

The participants said that teachers have great responsibilities, and their role requires, in addition to knowledge and clinical skills, other skills such as student management and communication skills, and the lack of any of these skills can cause problems. Moreover, working as a TA instead of being a student can cause anxiety due to the fear of facing unknown conditions. This category consists of four subcategories, including the stress of accepting the TA role, the lack of courses to familiarize TAs with their duties, insufficient supervision of the senior professor, and the chosen clinical field being different from the one the TA preferred. Each of these subcategories is described below.

### Stress of accepting the TA role

TAs were concerned with issues like the inability to answer students’ questions or communicate with students, as a TA mentioned:

“*In the first days, even though I had already studied the material, I was worried I might not answer students’ questions, and I was not prepared for how to behave in such a circumstance. In addition, I was not prepared to communicate with students.*” (P 4).

### The lack of courses to familiarize TAs with their duties

A primary concern of TAs was the lack of courses to familiarize them with TAs’ duties, responsibilities, teaching methods, and management skills. A participant said about the impact of the familiarization courses on the better performance of TAs:

“*Training courses or workshops should be held for people who want to become TAs to teach them some skills. These courses can help TAs perform their role better. In addition, the presence of the main teacher with the TA, at least for a few days, in addition to removing their stress, causes the TAs to learn some skill from them*.” (P 6).

### Inadequate supervision of senior professor

The participants said that senior professors as professors and clinical assistant supervisors play an essential role in guiding and training TAs. A participant said:

“*Unfortunately, my senior professor was too busy to answer my calls or messages. In addition, I was unable to see him when I went to his office, which made me frustrated. Students and TAs are often left alone during the semester. Moreover, the health center authorities do not monitor the method that TAs use to teach, convey material, give assignments, and evaluate*.” (P10).

### Inconsistency of the assigned clinical training field with what the TA prefers

Some TAs had worked in clinical departments as nurses, and they expected that they could select the clinical department that they preferred. Therefore, they were dissatisfied that their desire and experience were not being considered. For example, a participant said that:

“*My favorite field was the heart, and I had more studies and experience in that field. However, I had to attend ENT and dermatology departments, which had very specialized terms and procedures. As a result, my limited experience and knowledge in these fields increased my responsibility and worry*.” (P 1).

### Improper planning of the TA plan

The TA plan to employ post postgraduate students is an excellent opportunity for TAs to practice teaching as a teacher. However, if this plan is not well managed or planned, it faces TAs, students, and senior professors with essential problems and fails the plan to achieve its objectives. This main category consists of three subcategories: the lack of clear job descriptions for TAs, the interference of TAs’ clinical training programs with their education, and the lack of sufficient authority for TAs to make decisions, each described below.

### The lack of clear job descriptions for TAs

The participants suggested clarifying the activities they expect TAs to do for any task to remove ambiguity and confusion. A participant said:

“*TAs are unfamiliar with their duties and tasks. It is unclear what a TA is expected to do and to which extent he is allowed to do activities*.” (P 7).

### The interference of TAs’ clinical training programs with their education

TAs are often postgraduate nursing students who work as a TA in addition to their education. The participants said that TA plans do not consider their education and sometimes overlap and interfere with each other. A participant said:

“*Professors arrange TA plans for postgraduate students without considering their education schedules. I think it should be regulated to avoid overlaps, and TAs should be employed on days they have no educational programs to avoid challenges between TAs and the professors*.” (P 4).

### TAs lacking sufficient authority for decision-making

The participants said they should also be given enough authority to make independent decisions if given responsibilities. Sometimes, the lack of decision-making authority leads to barriers to students’ clinical training. A participant said:

“*If I could choose, I would never accept to be a TA because I am not given enough independence to make my own decisions. I prefer to be at the bedside as a clinical professor or nursing instructor rather than as a TA because, for example, I was allowed to go alone to give medicines to the patients. Many nurses say that we should be there and monitor the medicines*.” (P 5).

## Facilitators to fulfilling the TA role

Factors to facilitate the TA role have two main categories with five subcategories.

### Internal motivation

Intrapersonal motivation or internal motivation are some factors facilitating the TA role. The participants believed that factors like the desire to teach, strengthening internal abilities, and improving teaching skills facilitate the TA role. This category is divided into two subcategories: TAs’ interest in being a teacher and increased self-confidence.

### Interest in being a teacher

The participants mentioned that communicating with students and conveying educational concepts to them has been a pleasant experience, and they felt satisfied to convey their knowledge to others. A participant said:

“*I am interested in teaching, and I enjoy communicating with students and teaching them something they do not know. Easy teaching is one of my strengths, and I can educate students of any background. I considered it a debt to teach someone what I have learned, so I welcomed being a TA*.” (P 12).

### Increasing self-confidence

The participants mentioned that creating and strengthening the ability to transfer knowledge and experiences to students and make independent decisions in some educational situations are very important to them to have a sense of value and self-confidence. A participant said: “*Teaching undergraduate students causes me to have a great sense of value because I can transfer what I have learned in theoretical courses and acquired while working in the hospital to others. In addition, it is a practice of being independent for TAs*.” (P 10).

### Empowering TAs

Postgraduate students, especially those interested in teaching and training in the future, consider working as a TA an opportunity to learn teaching skills under the supervision and guidance of a professor and apply them in clinical education. They can ask questions from their responsible professor. However, they must study continuously to have up-to-date knowledge and skills. This category consists of three subcategories: practicing teaching skills, increasing theoretical and clinical knowledge, and perceived support from the senior professor.

### Practicing teaching skills

Teaching is an acquired skill that can be strengthened by practice. The participants consider being a TA an opportunity to practice communicating with students and personnel, student management methods, and clinical teaching skills. This is a precious opportunity for assistants who want to be a teacher in the future. A participant said:

“*I think if a TA spends more time studying and getting experience in the health center, he will become a better teacher. It would be better to study now, not when one has become a faculty member. Younger people have more energy to study, and it is better to gain good experiences, face teaching challenges, and learn how to interact with students when they are a TA*.” (P 2).

### Increasing theoretical and clinical knowledge

From the participants’ perspective, a good teacher should study continuously and use up-to-date resources. He should be superior concerning theoretical knowledge and clinical skills, and being a TA is an excellent opportunity to be prepared for being a professor. In this regard, a participant said:

“*Working as a TA increases knowledge, prevents forgetting information, makes the information up-to-date, and leads to continuous studies. For me, it was a positive process for education and increasing the level of my studies*.” (P 6). Another participant referred to the need to have information about clinical skills and being familiar with equipment:

“*A student working as a TA must have the necessary scientific and practical ability. To work in a clinical environment needs theoretical and practical abilities. Moreover, a TA should have the necessary knowledge and familiarity to work with the department’s tools and equipment and know how they work*.” (P 11).

### Perceived support from a senior professor

The participants mentioned that the presence of a senior professor and having a proper relationship with him could help them learn teaching skills and find an answer to their possible questions about teaching methods or managing conditions. In this regard, a participant said:

“*I think proper communication between teacher and TA can greatly facilitate this role because they can choose the content they want to teach to the bachelor students. That is, they can plan for the contents to be taught initially. It will be much better if they have a good relationship and can plan together*.” (P 10).

## Discussion

This qualitative study determines the barriers and facilitators to fulfilling the TA role from the nursing students’ perspective. The data analysis findings were divided into two main categories of barriers and facilitators to fulfilling the TA role. The barriers included not accepting the teaching assistant as a clinical instructor, not being prepared to accept the TA role, and improper planning of the TA plan. Studies show that the use of TAs in educational organizations and various situations and the importance of their role in education is increasing, and their proper use can improve the educational situation [16]. Existing studies report different results about the effect of the TA on learning, and where students do not welcome the TA, the effect of his role will decrease [17]. Our findings showed that some students did not welcome the TA role. The available evidence shows that the presence of TAs alongside the main professors effectively improves the quality of lesson planning and education, but TAs are not welcomed by all students. The concurrent presence of the TA and teacher can improve the students’ feedback. For example, sometimes the close age gap between the students and TAs makes them not follow TAs’ constructions, while sometimes this small age gap causes the students to move away from the main teacher and tend towards the assistants [18].

Among the barrier, according to our findings, was that the clinical department personnel were reluctant to cooperate with TAs, which indicate the non-acceptance of TAs by them. Existing studies show that TAs may be under various pressures, such as non-acceptance by staff and students and the non-cooperation of others with them [19, 20]. Although, despite these conditions, the use of TAs in educational institutions and clinical fields is always increasing [21], our results indicated that their role is associated with stress and anxiety. Other studies also show that TAs are under stress, mainly due to their lack of experience and lack enough knowledge about the situation [22]. In addition, the lack of courses to familiarize TAs with their duties is one of the most important reasons for their stress in their role [23]. Moreover, the lack of sufficient supervision of TAs’ performance is another challenge and barrier, which is consistent with the existing evidence [24]. However, monitoring the performance of TAs is very important for improving their performance [25] and working effectively [26].

Imposing a clinical field that TAs do not prefer and the interference of the TA training program with TAs’ education was another barrier. Existing studies show that the most suitable method for using TAs in teaching is where the selected field is the one that TAs are interested in [27]. Therefore, TAs’ interests and work experience must be considered in selecting TAs’ working fields [28]. Another barrier found in this study was inappropriate planning for TAs and the lack of specific job descriptions. The results of other studies also show that although the use of TAs is increasing, there is not a clear job description for them [29].

On the other hand, when a TA has sufficient knowledge and understanding of his duties and responsibilities, his work process will be facilitated, and he will bear less stress [30]. There is evidence that TAs’ familiarity with their duties is essential and significantly improves the quality of teaching by assistants [31]. In addition, the results indicate that neglecting the TAs’ class schedules and inconsistency of TAs’ working plan with their education results in interference and deteriorates strongly the quality of education for students and TAs, which is consistent with the results of other studies [32]. Among the barriers was that TAs did not have enough authority to make decisions. Studies show that understanding the role of TAs and the importance of their independence still needs serious investigations [33]. Employing TAs in educational situations under the supervision of main teachers is challenging because they lack independence and the authority to make decisions [29].

The facilitators of the TA’s role are divided into two general groups: internal motivation and empowerment of the TA. Other studies show that individual interest in accepting the TA role is an important factor for the success of the TA role [32] because individual interest plays an important role in facilitating the education process, and TA must be interested in teaching and education [34]. In addition, increasing self-confidence and acquiring the necessary knowledge and skills for teaching empower TAs in the education field, which is essential for the success of the TA role [35]. Working as a TA, on the one hand, is a suitable opportunity to acquire sufficient knowledge and teaching skills and become prepared to participate in educational fields, and on the other hand, increasing TAs’ self-confidence [36]. Moreover, the findings showed that the support of the senior professor and a proper relationship between the professor and TA facilitates learning teaching skills and teaching methods and management of conditions. Other studies have also referred to the importance of joint educational planning by the senior professor and TA. In addition, they have mentioned that the professor’s support in the educational fields for the assistants increases his self-confidence and improves the quality of education [35].

## Conclusions

Based on the findings of this study, the role of teacher assistants, along with significant strengths and opportunities for empowering and involving assistants in the educational field, faced important barriers. Therefore, attention to the role of teacher assistants and planning and implementing appropriate solutions to remove these barriers to the proper employment of assistants and the improvement of educational quality in various fields is essential.

Furthermore, to facilitate educational management processes and achieve better learning outcomes, appropriate planning is necessary for developing guidelines and written job descriptions related to teacher assistants and creating a suitable environment for their readiness and acceptance. It is also recommended to conduct and implement applied research to examine and create transparency in the issues and challenges related to teacher assistants. Developing proper plans and implementing applied research to investigate and clarify the TAs’ problems and challenges is also recommended.

### Supplementary Information

**Additional file 1.**

** Additional file 2.**

## Data Availability

The datasets used and/or analyzed during the current study are available from the corresponding author on request.
