# Supplementary material for: Barriers and facilitators to fulfilling the teaching assistant role from nursing students’ perspective: a qualitative study
Source: BMC Nurs. 2024 Jan 12;23:39. doi: 10.1186/s12912-023-01645-7 (PMC10785395; doi:10.1186/s12912-023-01645-7)
Supplement: Supplementary file 1 — Additional file 1. [file 12912_2023_1645_MOESM1_ESM.docx]

**Supplementary Material**

**Interview Questions**

- What is your opinion about TAs’ position?
- How would you describe being a TA?
- What problems have you experienced as a TA?
- Which conditions do you think to facilitate the TA role?
- What role do the senior professors play in the teaching assistant process?
- What suggestions do you have for changing the conditions of TA?
