# Supplementary material for: Barriers and facilitators to fulfilling the teaching assistant role from nursing students’ perspective: a qualitative study
Source: BMC Nurs. 2024 Jan 12;23:39. doi: 10.1186/s12912-023-01645-7 (PMC10785395; doi:10.1186/s12912-023-01645-7)
Supplement: Supplementary file 2 — Additional file 2. [file 12912_2023_1645_MOESM2_ESM.docx]

| Main Categories | | Sub Categories | Indexes |
| --- | --- | --- | --- |
| Facilitators to fulfilling the TA role | Empowering TAs | Practicing teaching skills | “I think if a TA spends more time studying and getting experience in the health center, he will become a better teacher. It would be better to study now, not when one has become a faculty member. Younger people have more energy to study, and it is better to gain good experiences, face teaching challenges, and learn how to interact with students when they are a TA.” (P 2) |
|  |  | Increasing theoretical and clinical knowledge | “Working as a TA increases knowledge, prevents forgetting information, makes the information up-to-date, and leads to continuous studies. For me, it was a positive process for education and increasing the level of my studies.” (P 6) |
|  |  | Perceived support from a senior professor | I think proper communication between teacher and TA can greatly facilitate this role because they can choose the content they want to teach to the bachelor students. That is, they can plan for the contents to be taught initially. It will be much better if they have a good relationship and can plan together.” (P 10) |
